# Prognostic value of NT-proBNP added to clinical parameters to predict two-year prognosis of chronic heart failure patients with mid-range and reduced ejection fraction – A report from FAR NHL prospective registry

**DOI:** 10.1371/journal.pone.0214363

**Published:** 2019-03-26

**Authors:** Jindrich Spinar, Lenka Spinarova, Filip Malek, Ondrej Ludka, Jan Krejci, Petr Ostadal, Dagmar Vondrakova, Karel Labr, Monika Spinarova, Monika Pavkova Goldbergova, Klara Benesova, Jiri Jarkovsky, Jiri Parenica

**Affiliations:** 1 Department of Cardiology, University Hospital Brno, Brno, Czech Republic; 2 Faculty of Medicine, Masaryk University, Brno, Czech Republic; 3 First Department of Internal Medicine, Cardiology and Angiology, St Anne’s University Hospital Brno, Brno, Czech Republic; 4 Department of Cardiology, Hospital Na Homolce, Prague, Czech Republic; 5 Institute of Pathological Physiology, Faculty of Medicine, Masaryk University, Brno, Czech Republic; 6 Institute of Biostatistics and Analyses, Faculty of Medicine, Masaryk University, Brno, Czech Republic; Scuola Superiore Sant'Anna, ITALY

## Abstract

**Background:**

According to guidelines, the prognosis of patients with chronic heart failure can be predicted by determining the levels of natriuretic peptides, the NYHA classification and comorbidities. The aim our work was to develop a prognostic score in chronic heart failure patients that would take account of patients’ comorbidities, NYHA and NT-proBNP levels.

**Methods and results:**

A total of 1,088 patients with chronic heart failure with reduced ejection fraction (HFrEF) (LVEF<40%) and mid-range EF (HFmrEF) (LVEF 40–49%) were enrolled consecutively. Two-year all-cause mortality, heart transplantation and/or LVAD implantation were defined as the primary endpoint (EP). The occurrence of EP was 14.9% and grew with higher NYHA, namely 4.9% (NYHA I), 11.4% (NYHA II) and 27.8% (NYHA III–IV) (p<0.001). The occurrence of EP was 3%, 10% and 15–37% in patients with NT-proBNP levels ≤125 ng/L, 126–1000 ng/L and >1000 ng/L respectively. Discrimination abilities of NYHA and NT-proBNP were AUC 0.670 (p<0.001) and AUC 0.722 (p<0.001) respectively. The predictive value of the developed clinical model, which took account of older age, advanced heart failure (NYHA III+IV), anaemia, hyponatraemia, hyperuricaemia and being on a higher dose of furosemide (>40 mg daily) (AUC 0.773; p<0.001) was increased by adding the NT-proBNP level (AUC 0.790).

**Conclusion:**

The use of prediction models in patients with chronic heart failure, namely those taking account of natriuretic peptides, should become a standard in routine clinical practice. It might contribute to a better identification of a high-risk group of patients in which more intense treatment needs to be considered, such as heart transplantation or LVAD implantation.

## Introduction

The prevalence of HF depends on the definition applied, but is at least 2% of the adult population in developed countries [1,2]. Apart from deterioration of the quality of life (as a result of poorer performance and repeated hospitalisations), chronic heart failure is prognostically significant, with 3-year all-cause mortality being approximately 25% [3,4].

Prognostic scoring systems are widely used and treatment of patients with specific cardiovascular diseases is based on their risk stratification; for example, the GRACE score is used for patients with acute coronary syndrome [5], the CHA2DS2-VASc score is used for patients with atrial fibrillation [6] etc. Moreover, according to the current European guidelines, the estimation of prognosis helps patients and physicians decide on the appropriate type and timing of therapies and contributes to a more efficient planning of health and social services and resources [1]. Risk scoring systems are only rarely used in patients with chronic heart failure, however. The NYHA Functional Classification and the level of natriuretic peptides are used most commonly in these patients. The current guidelines emphasise the prognostic significance of comorbidities in patients with chronic heart failure [1]. In 2016, we published a simple comorbidity scoring system called AHEAD for patients with acute heart failure. It is based on the patient’s age, atrial fibrillation, anaemia and renal insufficiency. Each comorbidity or age >70 years increased the one-year mortality rate by approximately 10% [7]. We formulated the hypothesis that comorbidities might have a similar prognostic significance for the stratification in the population of patients with stable chronic heart failure as well, and that they might also increase the prognostic significance of natriuretic peptide levels and of the NYHA classification.

The aim of our work was to describe the two-year prognosis of an unselected cohort of patients with stable systolic chronic heart failure who had either a reduced or a mid-range ejection fraction and to develop a prognostic score based on the NYHA classification, age, clinical parameters and the neurohumoral activation expressed by the NT-proBNP level.

## Methods

The study protocol complied with the Declaration of Helsinki and was approved by the Ethics Committee of the University Hospital Brno (Brno, Czech Republic). Written informed consents were obtained from all patients before their participation in the study.

The FAR NHL (FARmacology and NeuroHumoraL activation) registry is a database of stable patients treated in three departments providing specialised care of heart failure patients in the Czech Republic. Data on medical history, physical examination and biochemistry results including NT-proBNP were prospectively collected from November 2014 to November 2015. The primary endpoint, i.e. the two-year prognosis in terms of all-cause mortality, heart transplantation and/or left ventricular assist device (LVAD) implantation, was evaluated up to November 2017. Patients were followed up prospectively at outpatient departments and mortality rates were verified in the centralised database of the Ministry of Health of the Czech Republic. Monitored data on all patients were gathered at the end of the two-year follow-up.

Patients who had either heart failure with reduced ejection fraction (HFrEF) (LVEF<40%) or heart failure with mid-range ejection fraction (HFmrEF) (LVEF 40–49%) were eligible. The cohort included patients who were followed up and treated in cardiology departments for stable chronic heart failure; an attack of heart failure with an elevation of natriuretic peptides and a reaction to heart failure treatment was reported in medical history of all involved patients. Echocardiography was performed recently (i.e. within 12 months after the enrolment) in all patients involved in our study. Other structural and/or functional abnormalities related to heart failure were found in patients with LVEF between 40% and 49%: left ventricular hypertrophy (interventricular septum ≥ 11 mm or left ventricular mass index ≥ 115 g/m2 for men and ≥ 95 g/m2 for women), left atrial enlargement (left atrial volume index > 34 ml/m2) and/or diastolic dysfunction (with E/e' ≥13 and mean e' < 9 cm/s). The current NT-proBNP level <125 ng/L was not among exclusion criteria because all patients had increased levels of NT-proBNP in their medical history. Exclusion criteria were as follows: (1) not signing the informed consent, (2) signs and symptoms of acute decompensation of heart failure, and (3) condition other than heart failure that would certainly limit the mid-term prognosis of patients (e.g. advanced stage of cancer, severe dementia and others). Plasma levels of NT-ProBNP were analysed using the Cobas E411 NT-proBNP electrochemiluminescence immunoassay Kit (Elycsys proBNP II, Roche Diagnostics, Indianapolis, IN, USA). The level of blank was 3 ng/L, the level of detection was 5 ng/L, the measuring range was 5–35,000 ng/L, the functional sensitivity (i.e. the lowest analyte concentration that can be reproducibly measured with a coefficient of variation ≤20%) was 50 ng/L, and the cut-off value was 125 ng/L.

Standard descriptive statistics was applied in the analysis; continuous variables were described by mean ± SD and median (5th percentile; 95th percentile), whereas categorical variables were characterised by absolute and relative frequencies. Statistical significance of differences among groups of patients was analysed using the Kruskal-Wallis test for continuous variables and the Fisher’s exact test for categorical variables. The contribution of the NT-proBNP biomarker to the clinical model was evaluated according to previously published recommendations [8,9]. Logistic regression was adopted for the identification of predictors and the development of multivariate models of scoring systems and biomarkers; the models were evaluated using the Hosmer–Lemeshow test, C statistics and a reclassification analysis of model results. Time to combined endpoint was visualized using the Kaplan-Meier methodology and the computation of proportion of surviving patients and its 95% confidence interval in a given time point was based on Kaplan-Meier estimates, too. Statistical significance of differences in time to event among groups of patients was evaluated using the log-rank test. The analysis was performed using SPSS 24.0.0.1 (IBM Corporation, 2016) and R 3.5.1 with the PredictABEL package.

## Results

A total of 1,088 patients were included; their mean age was 64 ± 12 years and 80.9% of them were men (Table 1). Ischaemic heart disease was the most frequent cause of heart failure (50.1%), followed by dilated cardiomyopathy (41.6%). The median value of left ventricular ejection fraction (LVEF) was 30%. Beta-blockers were administered to 93.8% of patients, drugs blocking the renin-angiotensin system (ACE inhibitors and/or ARBs) were administered to 88.3% of patients and mineralocorticoid receptor antagonists (MRAs) were administered to 65.7% of patients. In summary, 143 patients (13.1%), 657 patients (60.4%) and 288 patients (26.5%) were classified as NYHA I, NYHA II and NYHA III or IV respectively. A decrease in functional capacity (according to NYHA) was associated with a decrease in systolic blood pressure, left ventricular ejection fraction and with a slight decrease in the haemoglobin level; by contrast, it was also associated with a slight increase in diastolic blood pressure, pulse rate and the serum level of creatinine. Patients with a higher NYHA class had more frequently diabetes mellitus and/or atrial fibrillation (Table 2). There was a strong relationship between the median levels of NT-proBNP and a higher NYHA class (152 ng/L for the NYHA I group, 503 ng/L for the NYHA II group, and 1,122 ng/L for the NYHA III–IV group; p<0,001).

**Table 1:** Basic characteristics of patients in the FAR NHL registry.

Parameter	Total (N = 1,088)
Sex–male	880 (80.9%)
Age	64 ± 12; 65 (40; 82)
BMI	29 ± 5; 28 (22; 39)
*Aetiology*	
IHD	545 (50.1%)
IHD+DCM	7 (0.6%)
DCM	453 (41.6%)
HCM	5 (0.5%)
Other	78 (7.2%)
SBP (mmHg)	129 ± 17; 128 (101; 160)
DBP (mmHg)	80 ± 10; 80 (61; 99)
Heart rate (min^-1^)	74 ± 13; 72 (55; 96)
LVEF (%)	31 ± 9; 30 (17; 45)
Atrial fibrillation	379 (34.8%)
Diabetes mellitus	422 (38.8%)
*NYHA classification*	
1	143 (13.1%)
2	657 (60.4%)
3–4	288 (26.5%)
Haemoglobin (g/l)	142 ± 16; 143 (113; 165)
Creatinine (μmol/l)	106 ± 54; 95 (65; 172)
NT-proBNP (ng/L)	1,375 ± 2,773; 511 (27; 4,886)
ACEI/ARB	961 (88.3%)
Beta-blockers	1020 (93.8%)
MRA (Verospiron)	715 (65.7%)
2-year mortality	132 (12.1%)
HTX within 2 years	24 (2.2%)
LVAD within 2 years	12 (1.1%)
2-year combined endpoint	162 (14.9%)

Continuous variables are described by mean ± SD and median (5th percentile; 95th percentile); categorical variables are characterised by absolute and relative frequencies.

BMI, body mass index; DBP, diastolic blood pressure; DCM, dilated cardiomyopathy; HCM, hypertrophic cardiomyopathy; IHD, ischaemic heart disease; LVEF, left ventricular ejection fraction; SBP, systolic blood pressure.

**Table 2:** Comparison of patients with different NYHA classes.

Parameter	NYHA 1 (N = 143)	NYHA 2 (N = 657)	NYHA 3–4 (N = 288)	P^1^
Sex–male	123 (86.0%)	530 (80.7%)	227 (78.8%)	0.192
Age	62 ± 12; 64 (37; 79)	65 ± 12; 66 (40; 83)	63 ± 12; 64 (43; 81)	**0.005**
BMI	28 ± 4; 28 (23; 37)	29 ± 5; 29 (22; 38)	29 ± 5; 28 (21; 40)	0.093
*Aetiology*				
IHD	70 (49.0%)	343 (52.2%)	139 (48.3%)	0.480
DCM	59 (41.3%)	273 (41.6%)	128 (44.4%)	0.679
Other	16 (11.2%)	45 (6.8%)	22 (7.6%)	0.210
SBP (mmHg)	133 ± 19; 130 (105; 170)	129 ± 17; 128 (102; 160)	126 ± 18; 125 (99; 158)	**< 0.001**
DBP (mmHg)	82 ± 12; 80 (65; 100)	80 ± 10; 80 (61; 97)	79 ± 11; 80 (60; 98)	**0.005**
Heart rate (min^-1^)	70 ± 13; 68 (53; 90)	73 ± 13; 72 (55; 96)	76 ± 12; 75 (60; 100)	**< 0.001**
LVEF (%)	36 ± 8; 38 (23; 47)	32 ± 8; 30 (19; 45)	26 ± 8; 25 (15; 42)	**< 0.001**
Atrial fibrillation	35 (24.5%)	228 (34.7%)	116 (40.3%)	**0.005**
Diabetes mellitus	28 (19.6%)	271 (41.2%)	123 (42.7%)	**< 0.001**
Haemoglobin (g/l)	147 ± 15; 148 (123; 167)	142 ± 15; 144 (114; 165)	138 ± 17; 138 (109; 163)	**< 0.001**
Creatinine (μmol/l)	95 ± 25; 91 (66; 134)	107 ± 59; 95 (65; 186)	109 ± 54; 99 (63; 176)	**0.003**
NT-proBNP (ng/L)	506 ± 920; 152 (13; 2 599)	1 232 ± 2 468; 503 (32; 4 251)	2 134 ± 3 714; 1 122 (62; 6 511)	**< 0.001**
ACEI/ARB	136 (95.1%)	585 (89.0%)	240 (83.3%)	**< 0.001**
Beta-blockers	130 (90.9%)	623 (94.8%)	267 (92.7%)	0.134
MRA (Verospiron)	66 (46.2%)	426 (64.8%)	223 (77.4%)	**< 0.001**
2-year combined endpoint	7 (4.9%)	75 (11.4%)	80 (27.8%)	**< 0.001**

Continuous variables are described by mean ± SD and median (5th percentile; 95th percentile); categorical variables are characterised by absolute and relative frequencies.

^1^ P-value of the Kruskal-Wallis test for continuous variables and P-value of the Fisher’s exact test for categorical variables are reported for the comparison of patients with different NYHA classes.

BMI, body mass index; DBP, diastolic blood pressure; DCM, dilated cardiomyopathy; IHD, ischaemic heart disease; LVEF, left ventricular ejection fraction; SBP, systolic blood pressure.

Overall, the all-cause two-year mortality was 12.1%; heart transplantation (HTx) and LVAD implantation were performed in 2.2% and 1.1% of patients respectively. A combined endpoint (mortality and/or HTx and/or LVAD implantation) occurred in 14.9% of patients.

### NYHA classification and prognosis

**Fig 1:**
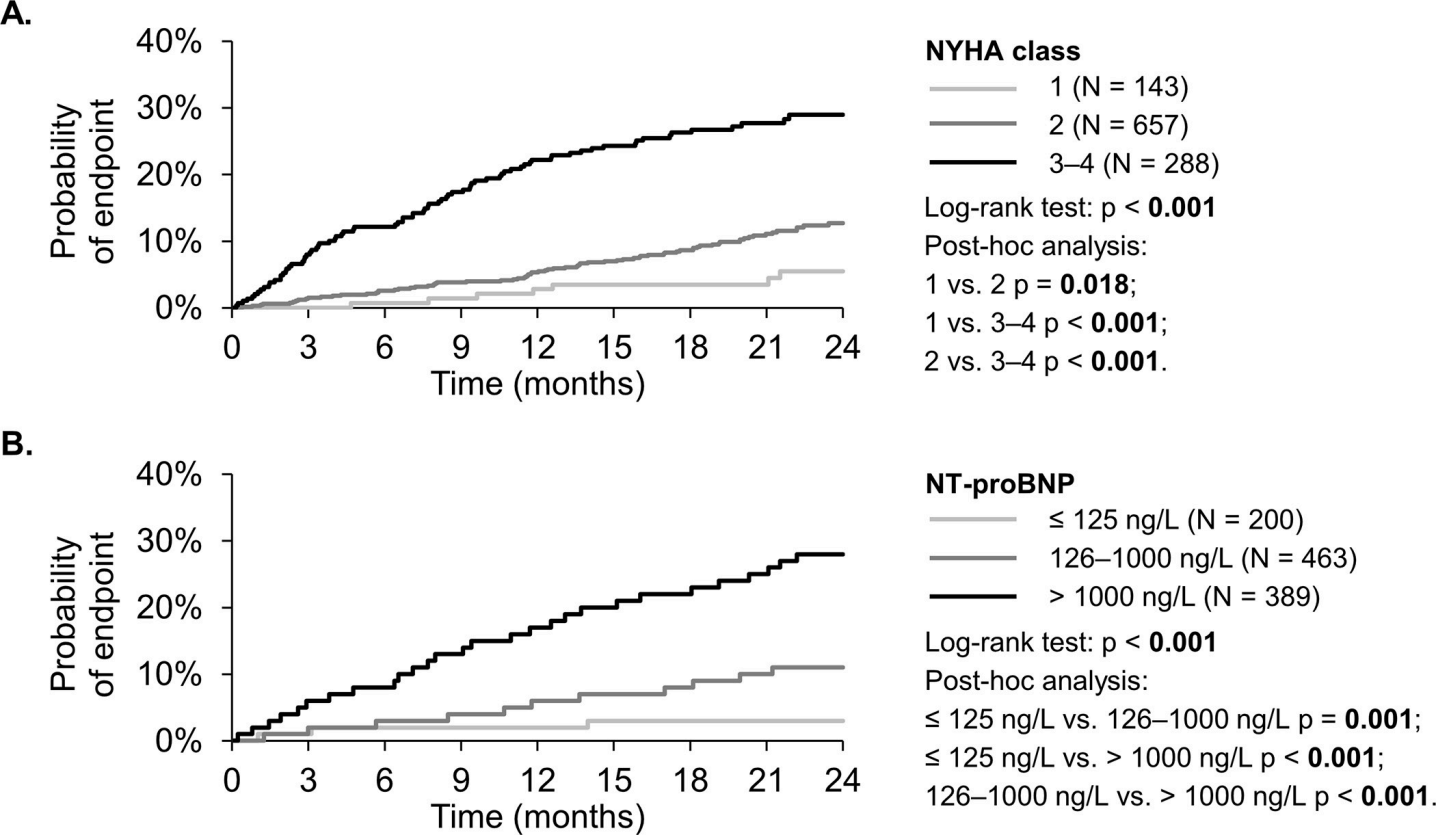
**2-year combined endpoint (death, HTX or LVAD).** A. by the NYHA class B. by the NTproBNP level.

The occurrence of combined endpoints was significantly different among groups of patients belonging to different NYHA classes, namely 4.9%, 11.4% and 27.8% (p < **0.001**) in groups of patients with NYHA I, NYHA II and NYHA III–IV respectively **(Fig 1A)**. C-statistic was used to calculate the AUC for the prediction of two-year prognosis according to the NYHA classification; in this way, the resulting AUC was 0.670 (p<0.001).

### NT-proBNP and prognosis

**Fig 2:**
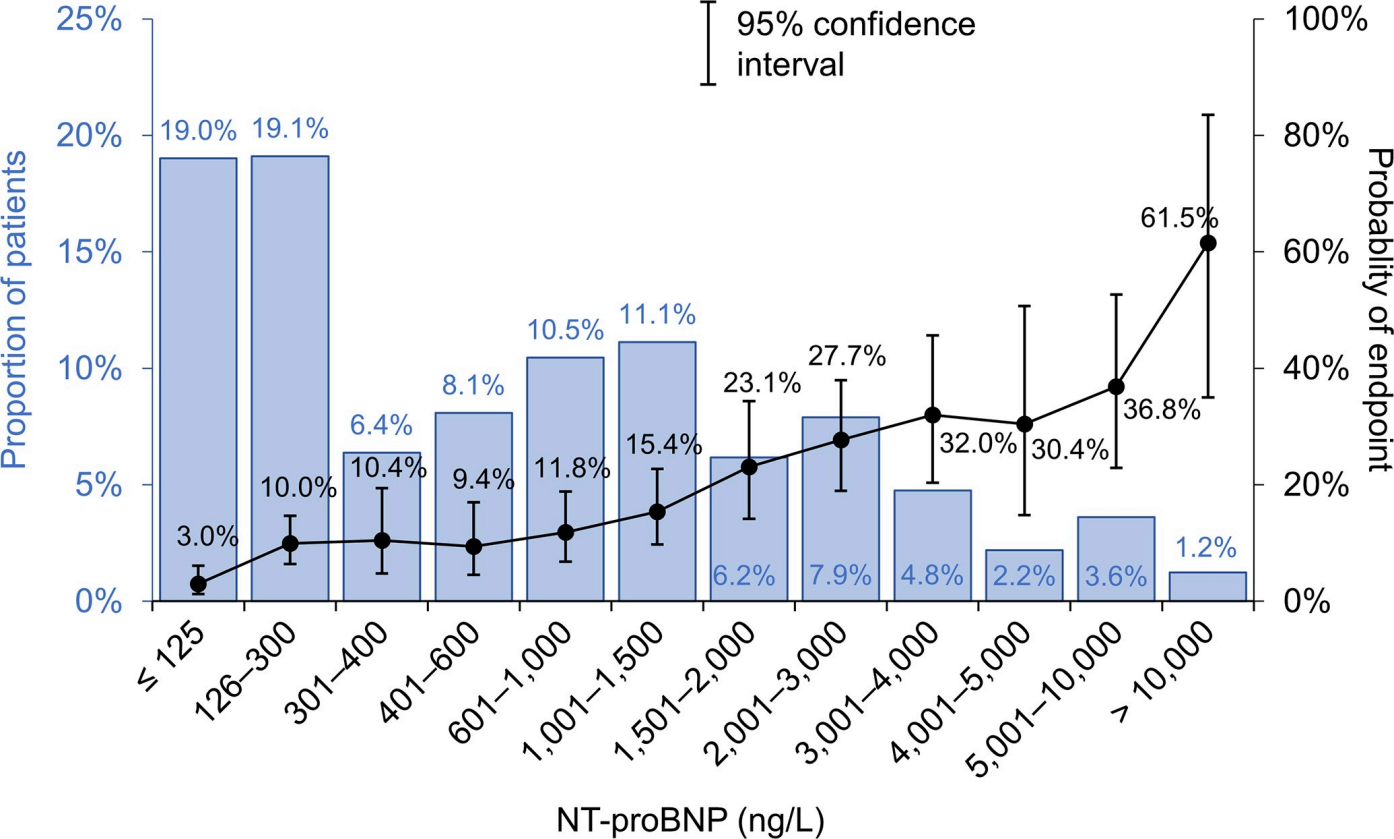
Distribution of NT-proBNP in patients with chronic heart failure and associated 2-year occurence of combined endpoint (death, HTX or LVAD).

**Fig 3:**
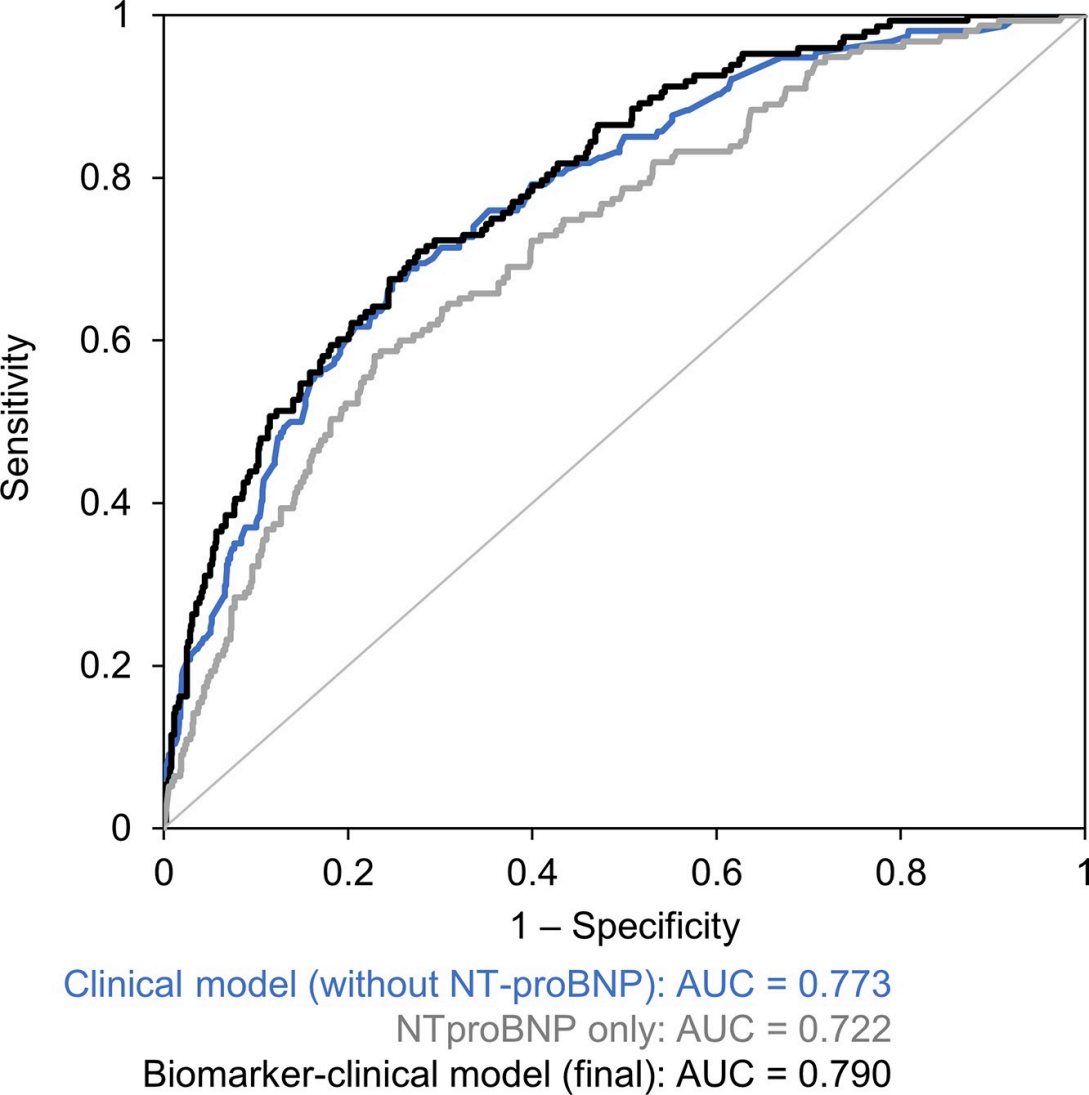
ROC curves for the prediction of primary endpoint (i.e. the two-year prognosis in terms of all-cause mortality, heart transplantation LVAD implantation).

The distribution of NT-proBNP levels in all patients in which a combined endpoint occurred is shown in Fig 2. The NT-proBNP level was ≤125 ng/L in 19.0% of patients and the occurrence of a combined endpoint was very low in this group (3.0%). The occurrence of a combined endpoint was approximately 10% in the groups of patients with NT-proBNP levels between 126 and 1,000 ng/L; by contrast, it grew from 15.4% to 36.8% in patients with NT-proBNP levels between 1,000 and 10,000 ng/L. Finally, the occurrence of a primary endpoint was 61.5% in the group of patients with NT-proBNP levels >10,000 ng/L, who accounted for only 1.2% of the entire cohort. Based on these results, we divided patients according to their NT-proBNP levels into three groups: ≤125 ng/L, 126–1,000 ng/L and >1,000 ng/L. Significantly different prognoses for these groups of patients are demonstrated by Kaplan-Meier curves (Fig 1B). The AUC for the prediction of two-year prognosis, based on the NT-proBNP level, was 0.722 (p<0.001) (Fig 3).

### Comorbidities, clinical and biochemical parameters and prognosis

Based on previously published prognostic models, we selected comorbidities, clinical characteristics, laboratory parameters and medication in which influence on the prognosis of patients with chronic heart failure had already been demonstrated (Table 3) [10–13]. We used univariate logistic regression to identify parameters for which the influence on the occurrence of a two-year combined endpoint was demonstrated in our cohort. Negative predictors included older age, COPD, advanced NYHA (III+IV), low systolic blood pressure (<110 mmHg), low LVEF (<35%), anaemia, high level of NT-proBNP, hyponatraemia, hyperuricaemia (>500 μmol/l), renal insufficiency (eGFR <60 ml/min) and, in terms of medication, the necessity of taking a higher dose of furosemide (>40 mg daily). On the other hand, higher diastolic pressure, higher cholesterol levels and tolerance/taking ACEI/ARB and beta-blockers were prognostically favourable.

**Table 3:** Univariate logistic regression models for the prediction of primary endpoint (i.e. the two-year prognosis in terms of all-cause mortality, heart transplantation and/or left ventricular assist device (LVAD) implantation).

		OR (95% CI)	P
**Anamnesis**			
Sex	Men (ref. women)	1.35 (0.86; 2.13)	0.197
Age (years)	10-unit increase	1.20 (1.04; 1.39)	**0.015**
	> 70 (ref. ≤ 70)	1.36 (0.96; 1.93)	0.083
BMI	> 30 (ref. ≤ 30)	0.80 (0.56; 1.14)	0.218
Diabetes mellitus	Yes (ref. no)	1.40 (1.00; 1.96)	0.052
Hypertension	Yes (ref. no)	1.21 (0.85; 1.74)	0.296
Smoking	Yes (ref. no)	1.27 (0.91; 1.77)	0.164
COPD	Yes (ref. no)	1.93 (1.26; 2.96)	**0.003**
NYHA	III+IV (ref. I+II)	3.37 (2.39; 4.75)	**< 0.001**
Heart rate (bpm)	> 90 (ref. ≤ 90)	1.36 (0.79; 2.34)	0.267
SBP (mmHg)	< 110 (ref. ≥ 110)	2.38 (1.53; 3.69)	**< 0.001**
	10-unit increase	0.79 (0.71; 0.88)	**< 0.001**
DBP (mmHg)	10-unit increase	0.63 (0.53; 0.75)	**< 0.001**
Ejection fraction (%)	< 35 (ref. ≥ 35)	2.12 (1.44; 3.11)	**< 0.001**
Atrial fibrillation	Yes (ref. no)	0.76 (0.44; 1.30)	0.312
LBBB	Yes (ref. no)	0.87 (0.59; 1.27)	0.466
Ischaemic etiology of heart failure	Yes (ref. no)	1.33 (0.95; 1.86)	0.095
Duration of heart failure (months)	> 18 (ref. ≤ 18)	1.26 (0.89; 1.79)	0.186
**Laboratory results**			
Hemoglobin (g/l)	< 130 in men, < 120 in women	2.70 (1.87; 3.89)	**< 0.001**
NT-proBNP (ng/L)	126–1,000 (ref. ≤ 125)	3.74 (1.57; 8.89)	**0.003**
	> 1,000 (ref. ≤ 125)	11.34 (4.88; 26.36)	**< 0.001**
Cholesterol (mmol/l)	< 3.1 (ref. 3.1–5.8)	1.37 (0.83; 2.27)	0.217
	> 5.8 (ref. 3.1–5.8)	0.47 (0.24; 0.93)	**0.030**
Natrium (mmol/l)	< 135 (ref. ≥ 135)	2.98 (1.77; 4.99)	**< 0.001**
Uric acid (μmol/l)	> 500 (ref. ≤ 500)	3.04 (2.09; 4.43)	**< 0.001**
eGFR (ml/min)	< 60 (ref. ≥ 60)	1.82 (1.29; 2.56)	**< 0.001**
**Medication**			
Furosemide (mg)	Yes (ref. no)	2.61 (1.50; 4.55)	**< 0.001**
	1–40 (ref. 0)	1.22 (0.67; 2.24)	0.515
	> 40 (ref. 0)	5.43 (3.06; 9.63)	**< 0.001**
Statins	Yes (ref. no)	1.06 (0.75; 1.50)	0.753
Betablockers	Yes (ref. no)	0.50 (0.28; 0.88)	**0.017**
ACEI/ARB	Yes (ref. no)	0.42 (0.27; 0.65)	**< 0.001**
Spironolacton/eplerenone	Yes (ref. no)	1.42 (0.97; 2.07)	0.070

**Fig 4:**
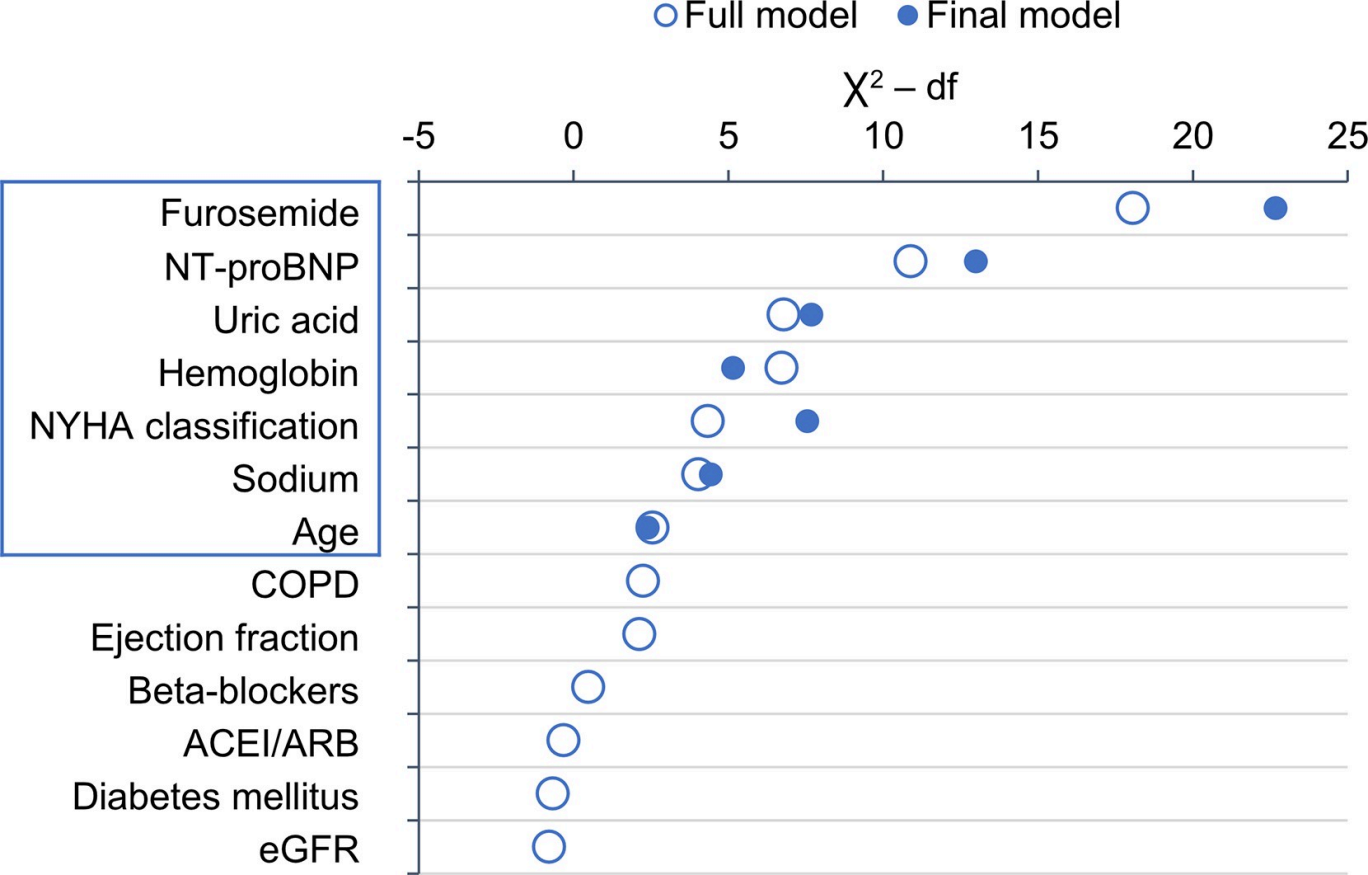
Importance of variables as measured by partial Wald χ2 minus the predictor degrees of freedom in the full model and in the final model selected by backward stepwise algorithm.

**Fig 5:**
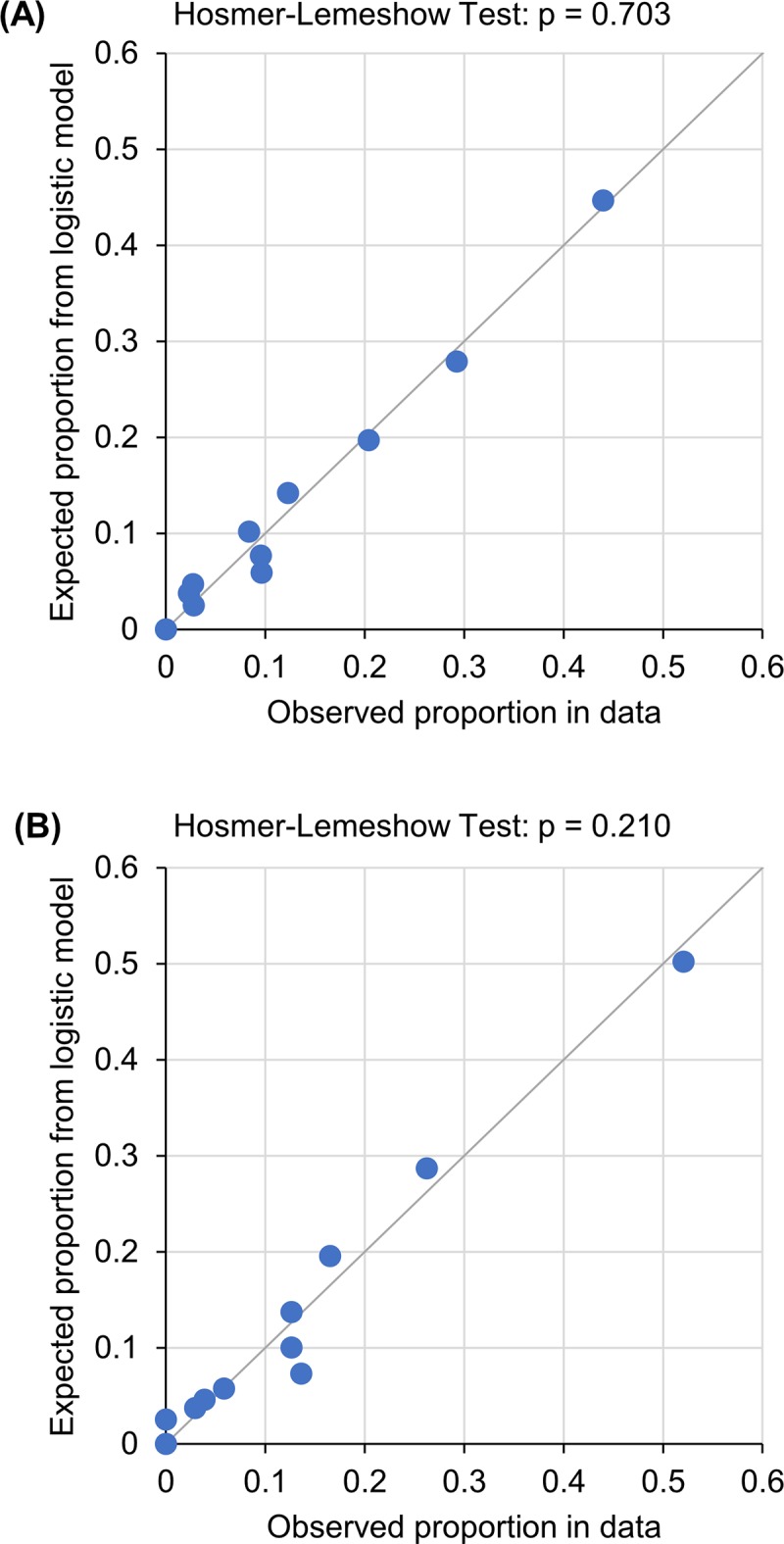
**Expected event rates (vertical axis) and observed event rates (horizontal axis), based on patients grouped into deciles of fitted risk values in (A) Clinical model and (B) Biomarker-clinical model**.

Multivariate logistic regression was used to develop a clinical model without NT-proBNP. Older age, advanced heart failure (NYHA III+IV), anaemia, hyponatraemia, hyperuricaemia and taking a higher dose of furosemide (>40 mg daily) were independent predictors (Table 4). The AUC of this clinical model for the prediction of the two-year endpoint was 0.773 (p<0.001) (Fig 3). Upon adding the new biomarker–the NT-proBNP level–to other clinical/biochemical parameters, multivariate logistic regression was used to develop a new model (a biomarker-clinical model); its predictive value, expressed by AUC and based on the C-statistic, was 0.790 (p<0.001). The significance of variables selected for the multivariate model is described in Fig 4. Adding more parameters to the above-mentioned selection of seven parameters did not lead to further improvements of predictive power of the model. Very good predictive powers of the developed models were validated by the Hosmer–Lemeshow test, which compared the predicted and observed frequency of a two-year combined endpoint for the clinical model (Fig 5A) and the final model (Fig 5B). A reclassification analysis confirmed the improvement of predictive power of the model after the addition of NT-proBNP when compared to the clinical model itself: the continuous free net reclassification improvement (cfNRI) was 0.330 (0.159;0.502) (p<0.001) and the integrated discrimination index was IDI 0.020 (0.006; 0.034) (p = 0.006).

**Table 4:** Multivariate logistic regression clinical model and model with NT-proBNP included for the prediction of primary endpoint (i.e. the two-year prognosis in terms of all-cause mortality, heart transplantation and LVAD implantation).

		Clinical model		Biomarker-clinical model	
Predictor		OR (95% CI)	P	OR (95% CI)	P
Age [years]	10-unit increase	1.23 (1.04; 1.46)	**0.015**	1.18 (0.99; 1.40)	0.065
NT-proBNP [ng/L]	1,000-unit increase	——		1.18 (1.08; 1.29)	**< 0.001**
NYHA classification	1-unit increase	1.92 (1.36; 2.72)	**< 0.001**	1.70 (1.19; 2.44)	**0.003**
Hemoglobin [g/l]	<130 in men, <120 in women	1.94 (1.07; 3.50)	**0.028**	1.73 (1.12; 2.67)	**0.013**
Sodium [mmol/l]	< 135 (ref. ≥ 135)	1.94 (1.07; 3.50)	**0.028**	2.07 (1.12; 3.81)	**0.020**
Uric acid [μmol/l]	100–unit increase	1.46 (1.20; 1.78)	**< 0.001**	1.36 (1.11; 1.67)	**0.003**
Furosemide [mg]	> 40 (ref. ≤ 40)	2.90 (1.94; 4.34)	**< 0.001**	2.84 (1.87; 4.33)	**< 0.001**

**Fig 6:**
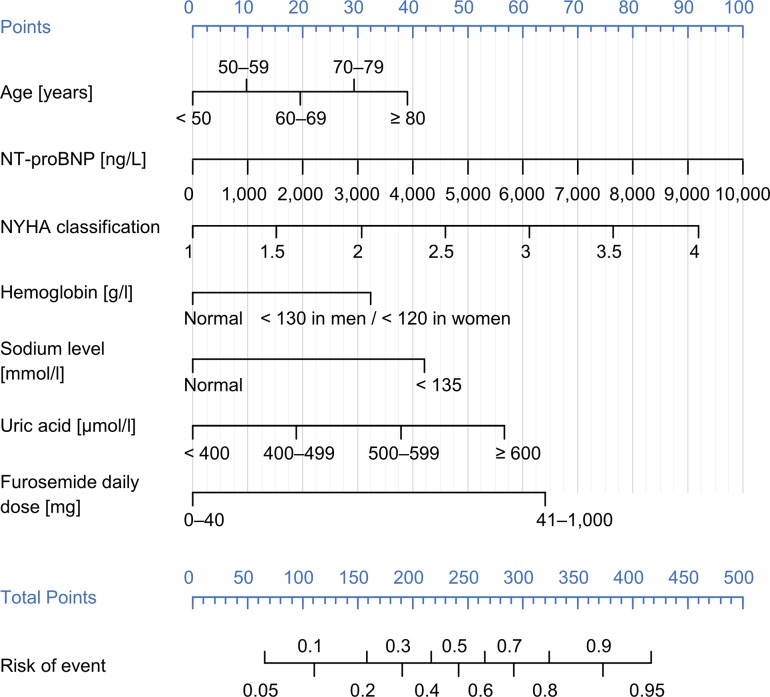
Nomogram of the proposed risk score (Biomarker-clinical model).

Results of the final model were visualised using a nomogram that describes the addition of individual variables to the overall score and the relation of the overall score of the model to the probability of endpoint occurrence in a given patient (Fig 6).

## Discussion

The paper presents four important results: (1) the NYHA classification represents a simple prognostic tool which, however, has a very limited predictive power. (2) The NT-proBNP level is a better simple prognostic tool. Our results suggest that patients can be divided into a very low-risk group (NT-proBNP level ≤ 125 ng/L, with 2-year all-cause mortality being about 3%), an intermediate-risk group (NT-proBNP level 126–1,000 ng/L, the endpoint occurrence being about 10%) and a high-risk group (NT-proBNP level >1,000 ng/L, with the risk growing from 15 to 61.5%). (3) Apart from the NYHA classification and older age, the developed clinical model highlights other comorbidities that are expressed mainly by laboratory parameters: anaemia, hyponatraemia and hyperuricaemia. Renal functions are expressed indirectly in the model, namely by the furosemide dose. The amount of furosemide dose depends on multiple factors, mainly on the level of congestion, renal function and the habitual practice of the attending physician [14]. (4) The NT-proBNP level brings another piece of information which can be added to the clinical model. The graphical representation of the model, based on a nomogram, provides a simple tool that can be readily used in clinical practice.

The plasma concentration of natriuretic peptides can be used as an initial diagnostic test in patients with dyspnoea to rule out the possibility of heart failure [1]. The upper limit of normal in the non-acute setting for NT-proBNP is 125 ng/L; in our cohort, we found NT-proBNP to be below this level in nearly 20% of patients with known HF, which is probably a sign of a very well compensated heart failure [15,16].

The New York Heart Association (NYHA) Functional Classification provides a simple way of classifying the extent of heart failure [17]. Despite difficulties in its application (such as the challenge of consistently classifying patients into class II or III), it remains arguably the most important prognostic marker for heart failure in routine clinical use. Most of the so far available clinical studies describe a correlation between the NYHA classification and NT-proBNP levels, a conclusion similar to our results. On the other hand, some of the other studies did not confirm this result [18].

From a translational point of view, the model highlights the prognostically most significant parameters. Although we cannot influence age in clinical practice, we might attempt to change other unfavourable parameters. More intense treatment might be considered at high NT-proBNP levels, whether it be pharmacological treatment, such as increasing the dose of diuretic drugs in order to achieve decongestion, titration of ACEI/ARB dose, exchanging ACEI/ARB for ARNI [19], or non-pharmacological treatment, such as revascularisation procedures in coronary artery disease [20], correction of valvular heart disease or cardiac resynchronisation therapy [1]. Although some analyses suggested that natriuretic peptide-guided treatment of chronic heart failure might have some benefits [21], a prospective clinical study did not confirm the benefit of such treatment [22]. Anaemia is linked to a poorer functional state and a poorer prognosis, and the cause of anaemia needs to be established [1]. The clinical trial of anaemia treatment in HFrEF patients using darbepoetin alfa did not show any improvement in the patients’ prognosis [23]. In cases of iron-deficiency anaemia in HFrEF patients, the functional capacity can be improved and the risk of rehospitalisation for heart failure can be decreased by intravenous iron therapy [24]. Hyperuricaemia is related to a poorer prognosis particularly in HFrEF patients [25,26]. Uric acid is an important part of the antioxidant system and it is not clear whether a decrease from a high level improves the patient’s prognosis or not. In the long term, high levels of uric acid might lead to kidney damage by uric acid nephropathy. In case of gout, the aim is to maintain a serum urate level below 357 μmol/l [1]. In case of hyponatraemia, restriction of fluids is imposed most frequently, followed by infusions of isotonic and hypertonic solutions of natrium and tolvaptan [27]. Unfortunately, treatment by tolvaptan, a vasopressin receptor antagonist which significantly increases natrium levels in patients with hyponatraemia, did not demonstrate any effect on the long-term mortality of HFrEF patients [28]. The dose of a loop diuretic corresponds to the severity of heart failure and patients should be treated with the lowest efficient dose of a loop diuretic that is sufficient to achieve well compensated and euvolemic state [14].

The predictive value of the biomarker-clinical model is very good when compared to previously published risk scores. The published results of discrimination ability described by the C-statistic for a one-year prediction of prognosis (MAGGIC with BNP/NTp-proBNP 0.736 [25], 3C-HF score 0.82–0.87 [26], BCN bio-HF calculator 0.79 [27]) or a two-year prediction of prognosis (Seatle Heart Failure Model 0.729 [28]) were comparable with our model (AUC 0.790).

Our study has a number of limitations. Only a population of patients with mid-range and reduced ejection fraction was described; patients with preserved ejection fraction were not included in the study. A high proportion of patients were treated with the combination of angiotensin-converting enzyme (ACE) inhibitors / angiotensin II receptor blockers (ARBs), beta-blockers and mineralocorticoid receptor antagonists (MRAs). At the time of data collection, angiotensin receptor-neprilysin inhibitors (ARNi) were not approved for therapeutic use in the Czech Republic; however, their use will be expanded soon, which is expected to have an impact on the level of natriuretic peptides, the NYHA class and the long-term prognosis. The newly developed model was not validated on another cohort of patients or cross-validated due to low sample size. The clinical benefit of use of the newly developed risk score must be prospectively validated in order to confirm benefits for the treatment of patients.

## Conclusion

NT-proBNP levels reflect the severity of stable chronic heart failure expressed by the NYHA Functional Classification. Very low levels of NT-proBNP are associated with an excellent prognosis, whereas high levels predict an unfavourable prognosis in the medium term. Taking NT-proBNP levels into consideration improves the discrimination value of a model based on clinical parameters. The prediction of prognosis for patients in clinical practice identifies the groups of high-risk patients who might benefit from care provided by specialised centres and from a more intense treatment of heart failure.

## Supporting information

S1 Table Data set used for the computation of the manuscript. (CSV)
